# Reference Data for the Ruff Figural Fluency Test Stratified by Age and Educational Level

**DOI:** 10.1371/journal.pone.0017045

**Published:** 2011-02-10

**Authors:** Gerbrand J. Izaks, Hanneke Joosten, Janneke Koerts, Ron T. Gansevoort, Joris P. Slaets

**Affiliations:** 1 University Center for Geriatric Medicine, University Medical Center Groningen, Groningen, The Netherlands; 2 Alzheimer Center Groningen, University Medical Center Groningen, Groningen, The Netherlands; 3 Department of Nephrology, University Medical Center Groningen, Groningen, The Netherlands; 4 Department of Clinical and Developmental Neuropsychology, University of Groningen, Groningen, The Netherlands; 5 Graduate School of Medical Sciences, University of Groningen, Groningen, The Netherlands; University of Granada, Spain

## Abstract

The Ruff Figural Fluency Test (RFFT) was developed to avoid the difficulties that were encountered in earlier tests of figural fluency. Although the test characteristics of the RFFT seem to be good and it is a valuable addition to neuropsychological assessments, reference data are still scarce. To this aim, we required 2,404 community dwelling persons in Groningen, the Netherlands to perform the RFFT. All 1,651 persons with a complete RFFT and known educational level formed the reference sample. Their age ranged from 35 to 82 years and their educational level from primary school to university grade. Ninety-six percent of the persons were of Western European descent. All tests were analyzed by two independent examiners and subsequently three measures were calculated: number of unique designs, number of perseverative errors and error ratio. The main finding was that performance on the RFFT was dependent on age and educational level. This was not only observed in older persons but also in young and middle-aged persons. Reference data for the three RFFT measures are presented in groups of five years of age ranging from 35–39 years to 75 years or older.

## Introduction

Executive functions are higher order cognitive processes that encompass skills necessary for purposeful, goal-directed behavior and are essential to the ability to respond to novel and unfamiliar situations [1], [2]. As patients with frontal lobe damage show marked changes in behavior, it is generally believed that the frontal lobes play an important role in executive functions. For that reason, executive functions are usually assessed with neuropsychological tests that can detect changes in frontal lobe function. As fluency tests are one type of test that consistently show defective performance in those with frontal lobe lesions [3], fluency tests are commonly used in clinical care to evaluate executive functions.

Fluency refers to the ability to maximize the production of responses under constraint of time and restricted search conditions while avoiding response repetition [4]. Fluency can be primarily measured as verbal fluency or figural (nonverbal) fluency. In verbal fluency tests, persons are required to produce as many words beginning with a specific letter or to name as many objects belonging to a specific category as possible within limited time. In figural fluency tests, persons are required to generate as many nonsense drawings or figures as possible within limited time [5]. The Design Fluency Test and the Five-Point Test are well known examples of figural fluency tests [6], [7]. These early tests have, however, some important disadvantages. The Design Fluency Test lacks clear scoring rules due to which examiners may reach different conclusions. The Five-Point Test has a relatively small scoring range due to which the test suffers from a ceiling effect and has a low sensitivity for subtle changes in frontal lobe function. Modifications of the Five-Point Test led to the development of the Ruff Figural Fluency Test (RFFT)[8].

The RFFT has well defined scoring rules and a wide range of scores and was investigated in various patient populations. Performance on the RFFT is, for example, associated with frontal gray matter volume in patients with Alzheimer's disease [9], and right frontal delta magnitude on quantitative electroencephalography [10]. In addition, performance on the RFFT is associated with diverse health conditions such as head injury [11], Alzheimer's disease [12], Parkinson's disease [12], and chronic alcohol abuse [13]. Because the test also has good test-retest reliability [8], and good to excellent interrater reliability [14], [15], the RFFT is a valuable addition to neuropsychological assessments in clinical settings. However, reference data of the RFFT are scarce and based on relatively small samples [8].

The aim of this paper was to provide reference data for the RFFT that are stratified by age, gender and educational level. The reference sample included 1,651 community dwelling persons who were aged 35 to 82 years, and had educational levels that ranged from primary school to university grade. The large reference sample allowed for the calculation of reference data for five-year age groups across a wide range of ages.

## Results

### Reference sample

The reference sample that included 1,651 persons comprised 47% men and 53% women (Table 1). Their mean age (SD) was 54 (11) years. Thirty-seven percent of the reference sample had a low educational level and 63% had a high educational level.

**Table 1:** Distribution of age, gender and educational level in the reference sample.

Age (years)	Educational level									
	Low (≤12 years)				High (>12 years)				All	
	Men		Women		Men		Women			
	N	%^a^	N	%^a^	N	%^a^	N	%^a^	N	%^a^
35–39	6	0.4	6	0.4	66	4.0	78	4.7	156	9.5
40–44	21	1.3	27	1.6	65	3.9	96	5.8	209	12.7
45–49	31	1.9	40	2.4	71	4.3	109	6.6	251	15.2
50–54	44	2.7	51	3.1	95	5.7	96	5.8	286	17.3
55–59	33	2.0	62	3.8	76	4.6	88	5.3	259	15.7
60–64	27	1.6	48	2.9	46	2.8	28	1.7	149	9.0
65–69	34	2.1	51	3.1	29	1.8	17	1.0	131	7.9
70–74	43	2.6	32	1.9	30	1.8	14	0.8	119	7.2
≥75	25	1.5	28	1.7	26	1.6	12	0.7	91	5.5
All	264	16.0	345	20.9	504	30.5	538	32.6	1651	100.0

a Percentage of the total reference sample (N = 1651). Sum of percentages may not equal percentage of All due to rounding.

The reference sample was slightly younger and had more often a high educational level than the persons who did not complete the RFFT. The gender distribution was similar in both groups. Persons who did not complete the RFFT (p-values for comparison with the reference sample) had a mean age (SD) of 56 (11) years (*p*<0.001). Fifty one percent of this group had a low educational level and 49% had a high educational level (*p*<0.001), 44% was male and 56% female (*p* = 0.28).

### Unique designs

**Figure 1:**
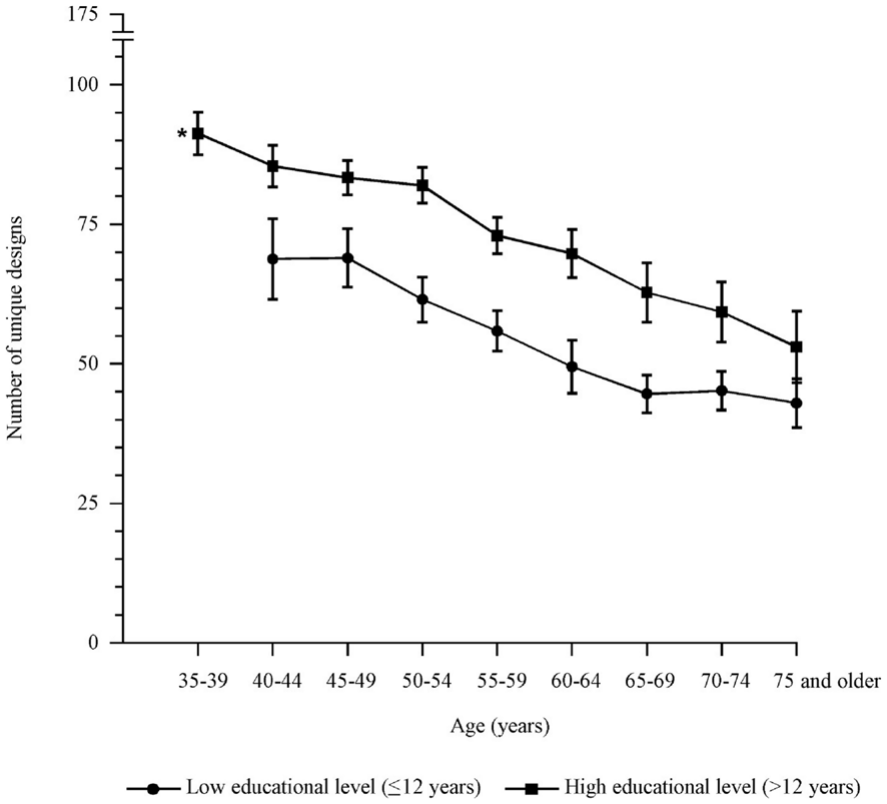
Mean number of unique designs dependent on age and educational level. *, only reported for persons with a high educational level as the number of persons with a low educational level was small in this age group. Bars represent 95% confidence intervals.

The number of unique designs was normally distributed in the reference sample with a mean (SD) of 70 (26) and it was clearly associated with age and educational level (Figure 1). The mean number of unique designs decreased with increasing age (Pearson's correlation coefficient, −0.50; 95%CI, 0.53 to −0.46; *p*<0.001). The decrease was −4.1 (95%CI, −4.9 to −3.4; *p*<0.001) per five years of age in persons with low educational level, and −4.6 (95%CI, −5.2 to 3.9; *p*<0.001) per five years of age in persons with high educational level. The mean number of unique designs in persons with low educational level was lower than in persons with high educational level: mean (SD), 55 (21) and 79 (24), respectively (*p*<0.001). This difference was found in all age groups (Figure 1). There was no difference between men and women: mean (SD), 70 (26) and 70 (25), respectively (*p* = 0.91). Reference data stratified for age and educational level are shown in Table 2 and Table 3.

**Table 2:** Number of unique designs for low educational level (≤12 years): percentile scores dependent on age.

Percentile	Age (years)								
	35–39	40–44	45–49	50–54	55–59	60–64	65–69	70–74	≥75
	N = 12	N = 48	N = 71	N = 95	N = 95	N = 75	N = 85	N = 75	N = 53
10	-a	40	41	34	33	29	25	24	23
20	-	49	47	43	42	32	30	30	29
30	-	52	53	51	45	39	35	39	33
40	-	55	60	57	51	42	40	43	36
50	-	65	65	62	54	46	43	45	40
60	-	74	76	67	59	50	45	48	45
70	-	81	84	71	65	54	49	52	52
80	-	88	90	78	72	67	56	57	53
90	-	101	102	88	80	75	70	66	68

a Not calculated because of the small number of persons.

**Table 3:** Number of unique designs for high educational level (>12 years): percentile scores dependent on age.

Percentile	Age (years)								
	35–39	40–44	45–49	50–54	55–59	60–64	65–69	70–74	≥75
	N = 144	N = 161	N = 180	N = 191	N = 164	N = 74	N = 46	N = 44	N = 38
10	57	50	57	52	47	42	44	36	25
20	71	67	66	62	52	49	48	43	38
30	82	76	73	69	60	62	52	45	43
40	86	82	78	75	65	66	57	52	47
50	93	86	83	82	72	70	59	57	52
60	98	91	88	88	79	75	63	65	57
70	104	96	94	95	85	81	65	71	59
80	111	104	102	102	90	85	80	76	63
90	121	114	111	110	102	94	93	83	79

### Perseverative errors

**Figure 2:**
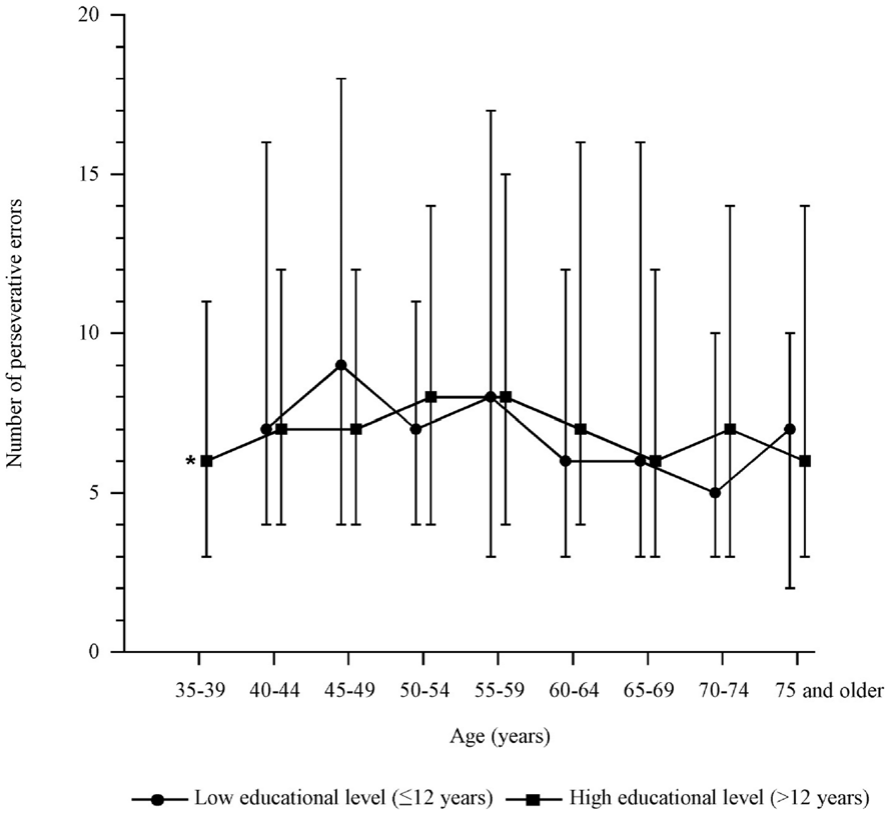
Median number of perseverative errors dependent on age and educational level. *, only reported for persons with a high educational level as the number of persons with a low educational level was small in this age group. Upper bars represent 75th percentile, lower bars represent 25th percentile.

The distribution of the number of perseverative errors in the reference sample was strongly skewed to the right with a median (interquartile range, IQR) of 7 (4 to 13). The difference in median number of perseverative errors between age groups was small and not statistically significant (*p* = 0.06) (Figure 2). The lowest median number (6 perseverative errors) was found in the age groups 35–39, 65–69 and 70–74 years and the highest median number (8 perseverative errors) was found in age group 55–59 years. There was no difference in number of perseverative errors between persons with low or high educational level: median (IQR), 7 (3 to 13) and 7 (4 to 13), respectively (*p* = 0.50). In addition, there was no difference between men and women: median number of perseverative errors (IQR), 7 (3 to 12) and 7 (4 to 15), respectively (*p* = 0.06). Reference data stratified for age are presented in Table 4.

**Table 4:** Perseverative errors for all educational levels: percentile scores dependent on age^a^.

Percentile	Age (years)									All
	35–39	40–44	45–49	50–54	55–59	60–64	65–69	70–74	≥75	
	N = 156	N = 209	N = 251	N = 286	N = 259	N = 149	N = 131	N = 119	N = 91	N = 1651
10	19	28	28	25	30	28	29	24	25	26
20	14	16	16	15	19	16	16	14	14	16
30	10	11	11	12	13	12	11	9	9	11
40	7	9	9	9	9	8	8	7	8	9
50	6	7	7	7	8	7	6	6	7	7
60	4	6	6	6	6	5	5	5	5	5
70	4	5	4	4	4	4	4	4	3	4
80	2	3	3	3	3	3	2	2	1	3
90	1	2	2	2	1	2	2	1	1	1

a Performance is better if the number of perseverative errors is lower.

### Error ratio

**Figure 3:**
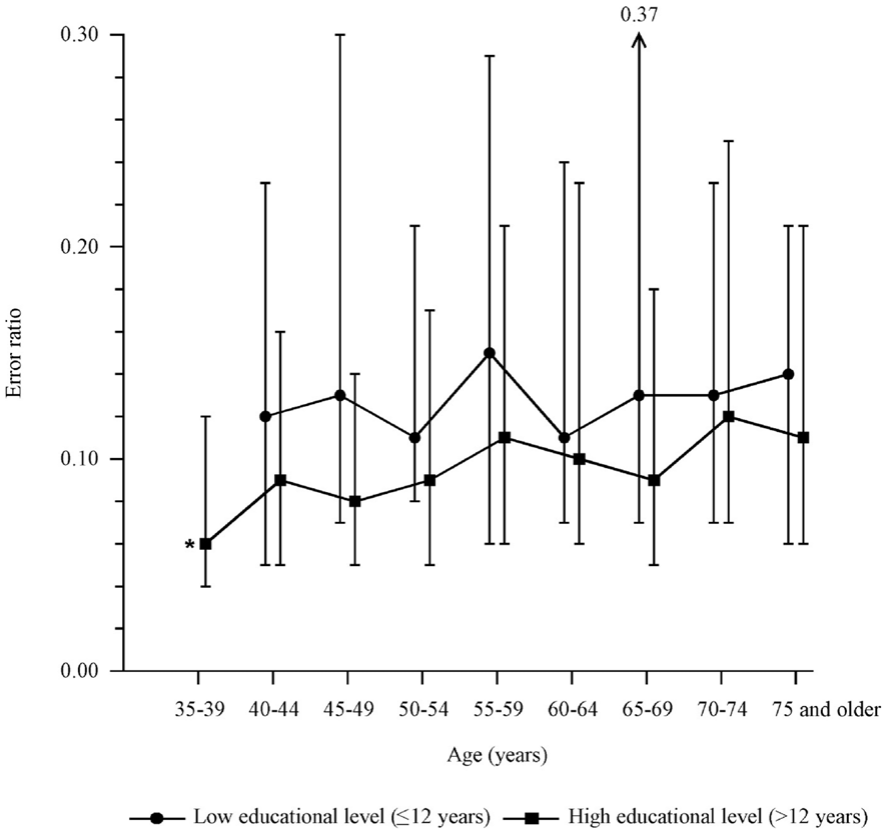
Median error ratio dependent on age and educational level. *, only reported for persons with a high educational level as the number of persons with a low educational level was small in this age group. Upper bars represent 75th percentile, lower bars represent 25th percentile. The difference between low and high educational level was statistically significant (*p*<0.001).

The distribution of the error ratio was also strongly skewed to the right. The median error ratio (IQR) was 0.10 (0.05–0.20). The error ratio was different between age groups and the median error ratio gradually increased from 0.06 at age 35–39 years to 0.13 at age 75 years and older (*p*<0.001) (Figure 3). Furthermore, there was a difference between educational levels. The median error ratio (IQR) was 0.13 (0.07 to 0.25) in persons with a low educational level and 0.09 (0.05 to 0.17) in person with a high educational level (*p*<0.001). There was no statistically significant difference between men and women: median error ratio (IQR), 0.10 (0.05 to 0.18) and 0.11 (0.5 to 0.21), respectively (*p* = 0.04). Reference data stratified for age and educational level are presented in Table 5 and Table 6.

**Table 5:** Error ratio for low educational level (≤12 years): percentile scores dependent on age^a^.

Percentile	Age (years)								
	35–39	40–44	45–49	50–54	55–59	60–64	65–69	70–74	≥75
	N = 12	N = 48	N = 71	N = 95	N = 95	N = 75	N = 85	N = 75	N = 53
10	-b	0.54	0.54	0.57	0.84	0.74	1.02	0.42	0.43
20	-	0.38	0.34	0.25	0.36	0.26	0.46	0.26	0.25
30	-	0.20	0.26	0.18	0.24	0.19	0.31	0.19	0.18
40	-	0.15	0.15	0.13	0.18	0.13	0.16	0.16	0.16
50	-	0.13	0.13	0.11	0.15	0.11	0.13	0.13	0.14
60	-	0.09	0.10	0.10	0.11	0.10	0.12	0.09	0.11
70	-	0.06	0.08	0.08	0.08	0.08	0.08	0.08	0.08
80	-	0.04	0.05	0.06	0.05	0.05	0.06	0.06	0.04
90	-	0.02	0.02	0.03	0.02	0.04	0.04	0.02	0.03

a Performance is better if the error ratio is lower.

b Not calculated because of the small number of persons.

**Table 6:** Error ratio for high educational level (>12 years): percentile scores dependent on age^a^.

Percentile	Age (years)								
	35–39	40–44	45–49	50–54	55–59	60–64	65–69	70–74	≥75
	N = 144	N = 161	N = 180	N = 191	N = 164	N = 74	N = 46	N = 44	N = 38
10	0.21	0.34	0.27	0.30	0.37	0.43	0.25	0.47	0.78
20	0.14	0.19	0.16	0.20	0.26	0.30	0.23	0.34	0.27
30	0.10	0.14	0.12	0.14	0.19	0.19	0.15	0.19	0.21
40	0.09	0.11	0.10	0.11	0.14	0.14	0.11	0.15	0.17
50	0.06	0.09	0.08	0.09	0.11	0.10	0.09	0.12	0.11
60	0.05	0.07	0.06	0.08	0.08	0.09	0.08	0.10	0.09
70	0.04	0.05	0.05	0.06	0.06	0.08	0.07	0.07	0.08
80	0.03	0.04	0.04	0.04	0.05	0.05	0.04	0.05	0.05
90	0.01	0.02	0.02	0.03	0.02	0.03	0.02	0.03	0.02

a Performance is better if the error ratio is lower.

### Comparison with US reference sample

The mean number of unique designs in the PREVEND reference sample was lower than in the US reference sample. The difference varied between 10 and 20 unique designs (Table S1). The mean number of perseverative errors in the PREVEND reference sample was higher than in the US reference sample (with exception op persons aged 40–54 years and ≥16 years of education). The difference varied between one and four perseverative errors (Table S2). Thus, in general, the performance on the RFFT in the PREVEND reference sample was lower than in the US reference sample.

## Discussion

In this paper, we present reference data for the RFFT. These reference data have two strengths. Firstly, they were based on data from a large community based cohort whereas up to now, reference data for the RFFT were based on relatively small samples. The reference data in the professional manual of the RFFT, for example, were based on a US reference sample that included 358 persons [16]. Therefore, it may be assumed that the reference data from this study are more precise. Secondly, the reference sample of this study included a considerable number of older persons. In the professional manual, reference data are available up to the age of 70 years. Here, we also present reference data for persons aged 70 to 74 years and persons aged 75 years and older. Adding these age groups is useful because the number of highly aged individuals in clinical practice will steeply increase in the following years.

Comparable with previous studies [8], [16], performance on the RFFT was dependent on age and educational level. The number of unique designs decreased with increasing age and was higher in persons with high educational level compared with persons with low educational level. Similar results have been reported for other figural fluency tests [17]. As in previous studies, the association between the number of perseverative errors and age and educational level was not clear. The error ratio, however, was also associated with age and education. It increased with increasing age, and in all age groups, the error ratio was lower in persons with a low education level. Interestingly, the changes in performance on the RFFT could also be observed in the youngest age groups. Performance decreased with every five-year increment from the age of 35 years. Thus, the RFFT is sensitive to changes in cognitive function in young and middle-aged persons. Although this characteristic may not be relevant in clinical practice, it is highly valuable in large observational studies into the mechanisms of cognitive decline and dementia.

In contrast to the overall picture, the number of unique designs did not decline in persons with a low educational level who were aged 65 years or older. This could be due to a selective drop out of older persons with a low educational level and significant decline of cognitive function. Consequentially, the calculated reference data in this age group may be too high.

In general, performance on the RFFT in our reference sample was lower than in the US reference sample of the professional manual [8], [16]. Particularly, the differences in the number of unique designs were large. These difference can be explained in several ways. Firstly, the reference data of the professional manual were based on a small sample. As a consequence, these reference data probably are not precise. Secondly, whereas persons with diseases were excluded from the US reference sample of the professional manual [8], they were not excluded from the present reference sample nor were persons with extreme scores (outliers). Therefore, it is likely that the health status of the present reference sample is worse but also a better reflection of the health status of the general population. This is important because the main aim of reference data is to compare the performance of an individual with the performance of the general population. Finally, there may be educational but also cross-cultural differences between the samples. When neuropsychological test performance in different cultural groups is compared, significant differences are evident. This was not only found for verbal tests but also for nonverbal tests [18]. Performance on the Five-Point Test [7], for example, was negatively associated with age in a German reference sample but not in an Arabic reference sample [17], [19]. Therefore, the findings of this study may only apply to populations of Western European descent.

In conclusion, performance on the RFFT was dependent on age and educational level but not on gender. This was not only observed in older persons but also in young and middle-aged persons. Therefore, reliable reference data that are based on large study samples are necessary. However, reference data should be used with caution in populations from different cultural background.

## Materials and Methods

The data of the study were derived from the third survey of the Prevention of Renal and Vascular ENd-stage Disease (PREVEND) study. The PREVEND study was designed to investigate prospectively the natural course of microalbuminuria and its relation to renal and cardiovascular disease in the general population. Details of the PREVEND study protocol have been published elsewhere [20], [21], and can be found at www.prevend.org.

### Study Population and Reference Sample

The study population included 2,404 participants of the PREVEND study who were randomly selected from the general population (the so-called Groningen Random Sample) and completed the third survey of the PREVEND study. All persons were inhabitants of the city of Groningen, the Netherlands, and aged 35 to 82 years. Ninety-six percent of the persons was of Western European descent. A total of 1,665 persons completed the RFFT (69%) of whom 14 persons (1%) were excluded because their educational level was not known. Thus, the reference sample included 1,651 persons (68%).

The analysis was limited to the Groningen Random Sample because, due to its design, the total PREVEND cohort comprised a relatively high number of persons with microalbuminuria which is an established cardiovascular risk factor and probably negatively associated with cognitive function [22], [23]. The prevalence of microalbuminuria in the Groningen Ramdom Sample was similar to the prevalence in the general population (8%).

Persons who were unwilling or unable to participate were excluded. There were no other exclusion criteria and all participants of the third survey of the PREVEND study were required to perform the test.

### Procedure and Materials

All participants were required to visit the PREVEND outpatient department twice. During these visits, trained personnel assessed demographic, anthropometric, and cardiovascular risk factors and obtained blood samples for the measurement of hematological and biochemical parameters. The Ruff Figural Fluency Test was performed at the second of the two visits.

### Ruff Figural Fluency Test

**Figure 4:**
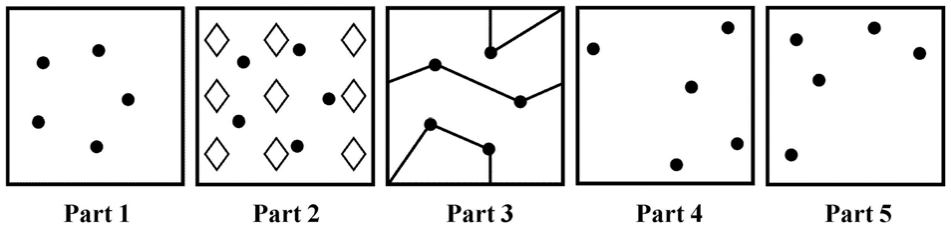
Five-dot patterns in parts 1 to 5 of the Ruff Figural Fluency Test [8]. Each part consists of 35 identical five-dot patterns.

The Ruff Figural Fluency Test (RFFT) is a measure of nonverbal fluency which has five parts (Figure 4) [2], [8], [16]. All parts (1 to 5) consist of 35 five-dot patterns arranged in seven rows and five columns on a 8.5×11″ sheet of paper. However, the stimulus pattern differs between the parts. In part 1, the five-dot pattern forms a regular pentagon. In parts 2 and 3, the five-dot pattern of part 1 is repeated but includes various distractors: diamonds in part 2, and lines in part 3. In parts 4 and 5, the five-dot pattern is a variation of the pattern of part 1 and these parts do not contain distracting elements. In each part, the task is to draw as many unique designs as possible within one minute by connecting the dots in a different pattern. Repetitions of designs are scored as perseverative errors. Performance on the RFFT is expressed as the total number of unique designs (the sum of all five parts), the total number of perseverative errors and the error ratio that is calculated by dividing the total number of perseverative errors by the total number of unique designs [2], [16].

In the PREVEND study, performance on the RFFT was analyzed independently by two trained examiners. The analysis was repeated by a third independent examiner if the number of unique designs or perseverative errors as analyzed by the first two examiners differed by more than two points in one part or more than four points in total. Then, for each participant, the RFFT scores as analyzed by the two examiners who were most concordant, were averaged and rounded to the nearest integer. This was done because generally the scoring rules of the RFFT allow for small variation between examiners. The averaged and rounded RFFT scores were used to calculate the reference data.

The concordance between the two ratings that were used to calculate the RFFT score for each participant was high.The mean difference (SD) between the ratings was 0.1 (1.8) for the total number of unique designs, and 0.1 (1.6) for the total number of perseverative errors. The Intraclass Correlation Coefficient (95% confidence interval) between the two ratings was 1.00 (0.99 to 1.00) for the total number of unique designs as well as for the total number of perseverative errors.

### Age

Age groups in five-year increments were defined by the age of the participants on the date they performed the RFFT. The following age groups were created: 35 to 39 years, 40 to 44 years, 45 to 49 years, 50 to 54 years, 55 to 59 years, 60 to 64 years, 65 to 69 years, 70 to 74 years, and 75 years and older.

### Educational Level

Educational level was divided into two groups according to the International Standard Classification of Education (ISCED)[24]. Low educational level corresponded to ISCED 0 to 2 (≤12 years of education). High educational level corresponded to ISCED 3 to 5 (>12 years of education).

### Comparison with US Reference Sample

Performance on the RFFT in the PREVEND reference sample was compared to the performance in the US reference sample [8], [16]. To enable an exact comparison, age groups and educational levels were defined according to the US reference sample: age groups, 40–54 years and 55–70 years; educational levels, ≤12 years (ISCED 0–2), 13–15 years (ISCED 3–4), and ≥16 years (ISCED 5). The comparison was only made for the total number of unique designs and the total number of perseverative errors as raw data on the error ratio were not available for the US reference sample [8].

### Ethics Statement

The PREVEND study has been approved by the Medical Ethical Committee (METc) of the University Medical Center Groningen and is conducted in accordance with the guidelines of the Declaration of Helsinki. Written informed consent was obtained from all participants.

### Statistical Methods

Data are presented as mean and standard deviation (SD) or 95% confidence interval (CI) if their distribution was normal. Otherwise they are presented as median and interquartile range (IQR). The difference in age between persons who did complete the RFFT and persons who did not complete the RFFT was tested with the independent-samples t test and the difference in educational level and gender distribution between these groups was tested with the chi-square test. The association between age and the number of unique designs was calculated as Pearson's correlation coefficient. The decrease in the number of unique designs per age group was estimated with linear regression analysis (regression model, number of unique designs  = a+b x age group). Differences in the number of unique designs between persons with low and high educational level and between men and women were also tested with the independent-samples t test. The differences in the number of perseverative errors and the error ratio between age groups, between low educational level and high educational level, and between men and women were tested with the Mann-Whitney U test or, if appropriate, Kruskal-Wallis H test. Percentiles were calculated in 10%-increments. Statistical analysis was done with SPSS 16.0 for Windows (SPSS Inc., Chicago, IL). Because a large reference sample may lead to overinterpretation of small differences between subgroups, the significance level was set at 0.01 instead of 0.05.

## Supporting Information

Table S1 Comparion of performance on the RFFT between the PREVEND reference sample and the US reference sample: unique designs.
(DOC)

Table S2 Comparion of performance on the RFFT between the PREVEND reference sample and the US reference sample: perseverative errors.
(DOC)
