# Supplementary material for: Reference Data for the Ruff Figural Fluency Test Stratified by Age and Educational Level
Source: PLoS One. 2011 Feb 10;6(2):e17045. doi: 10.1371/journal.pone.0017045 (PMC3037396; doi:10.1371/journal.pone.0017045)
Supplement: Table S1 — Comparion of performance on the RFFT between the PREVEND reference sample and the US reference sample: unique designs. (DOC) [file pone.0017045.s001.doc]

| **Education (years)** | **Age (years)** | **PREVEND reference sample^a^** | | | **US reference sample^b^** | | |
| --- | --- | --- | --- | --- | --- | --- | --- |
|  |  | **N** | **Mean** | **SD** | **N** | **Mean** | **SD** |
| ≤12 | 40-54 | 194 | 66 | 23 | 18 | 86 | 22 |
|  | 55-70 | 255 | 50 | 19 | 27 | 68 | 20 |
| 13-15 | 40-54 | 198 | 77 | 23 | 32 | 94 | 18 |
|  | 55-70 | 123 | 67 | 19 | 31 | 77 | 18 |
| ≥16 | 40-54 | 297 | 89 | 21 | 32 | 106 | 16 |
|  | 55-70 | 161 | 73 | 21 | 27 | 84 | 21 |

^a^ Groningen, the Netherlands.

^b^ Ruff RM, Light R, Evans R (1987) The Ruff Figural Fluency Test: a normative study with adults. Dev Neuropsychol 3: 37-51; Ruff RM (1996) Ruff Figural Fluency Test: professional manual. Lutz: Psychological Assessment Resources, Inc.
